# TfR1 deficiency beyond combined immunodeficiency. Pivotal role of mitochondrial function and iron-sulfur cluster biogenesis in disease pathogenesis

**DOI:** 10.1016/j.gendis.2026.102094

**Published:** 2026-02-19

**Authors:** Gerard Muñoz-Pujol, Elise J. Huisman, Juan-Francisco Mesa, Marieke Joosten, Annick Devos, Miguel Fernandez-Burriel, Olatz Ugarteburu, Eulàlia Segur-Bailach, Sabrina Gea-Sorlí, Sonia Moliner, Laia Farré-Tarrats, Mariona Guitart-Mampel, Gianluca Arauz-Garofalo, Marina Gay, Neus Villamor, Gemma Martín, María Calvo, Gloria Garrabou, María J. Macias, Laura Gort, Judit García-Villoria, Cristina Fillat, Frederic Tort, Antonia Ribes

**Affiliations:** a Section of Inborn Errors of Metabolism-IBC, Department of Biochemistry and Molecular Genetics, Hospital Clinic de Barcelona, Barcelona 08028, Spain; b Biomedical Research Institute August Pi i Sunyer (IDIBAPS), Barcelona 08036, Spain; c Biomedical Network Research Centre on Rare Diseases (CIBERER), Instituto de Salud Carlos III, Barcelona 08036, Spain; d Research Unit of Genomic Medicine, UAB-IR Sant Pau, Barcelona 08041, Spain; e Department of Pediatric Hematology, Erasmus MC Sophia Children's Hospital, University Medical Center Rotterdam, Rotterdam, CA 3000, the Netherlands; f Department of Pediatrics, Merida Hospital, Mérida 06800, Spain; g Department of Clinical Genetics, Erasmus MC Sophia Children's Hospital, University Medical Center Rotterdam, Rotterdam, CA 3000, the Netherlands; h Department of Pediatric Radiology, Erasmus MC Sophia Children's Hospital, University Medical Center Rotterdam, Rotterdam, CA 3000, the Netherlands; i Department of Clinical Chemistry, Mérida Hospital, Mérida 06800, Spain; j Inherited Metabolic Diseases and Muscular Disorders Reserach Lab, Cellex, Barcelona 08036, Spain; k Faculty of Medicine and Health Science - University of Barcelona (UB), Barcelona 08036, Spain; l Institute for Research in Biomedicine (IRB Barcelona), The Barcelona Institute of Science and Technology, Barcelona 08028, Spain; m Unitat d’Hematopatologia, Servei d’Anatomia Patològica, Hospital Clínic de Barcelona, CIBERONC, ISCIII, Barcelona 08036, Spain; n Advanced Optical Microscopy Facility, Scientific and Technological Centers, University of Barcelona, Barcelona 08036, Spain

*TFRC* encodes TfR1, a plasma membrane protein essential for cellular iron uptake.^1^ The pathophysiological consequences of mutations in this gene remain incompletely understood, and the role of TfR1 protein in iron homeostasis has largely been inferred from knockdown studies in cultured cells and animal models.^1^^,^^2^ The few reported patients with mutations in this gene have been exclusively linked to combined immunodeficiency (CID) (MIM #616740) and to unique mutations affecting the internalization motif of the protein.^3^, ^4^, ^5^

We report three individuals from two unrelated families with recessive biallelic *TFRC* variants (Fig. 1A) that do not present with CID but instead exhibit lethal hypertrophic cardiomyopathy and severe microcytic anemia as the predominant clinical symptoms, respectively. In both families, variants were validated by Sanger sequencing (Fig. S1A) and initially classified as variants of uncertain significance (VUS) (Table S1).

**Figure 1:**
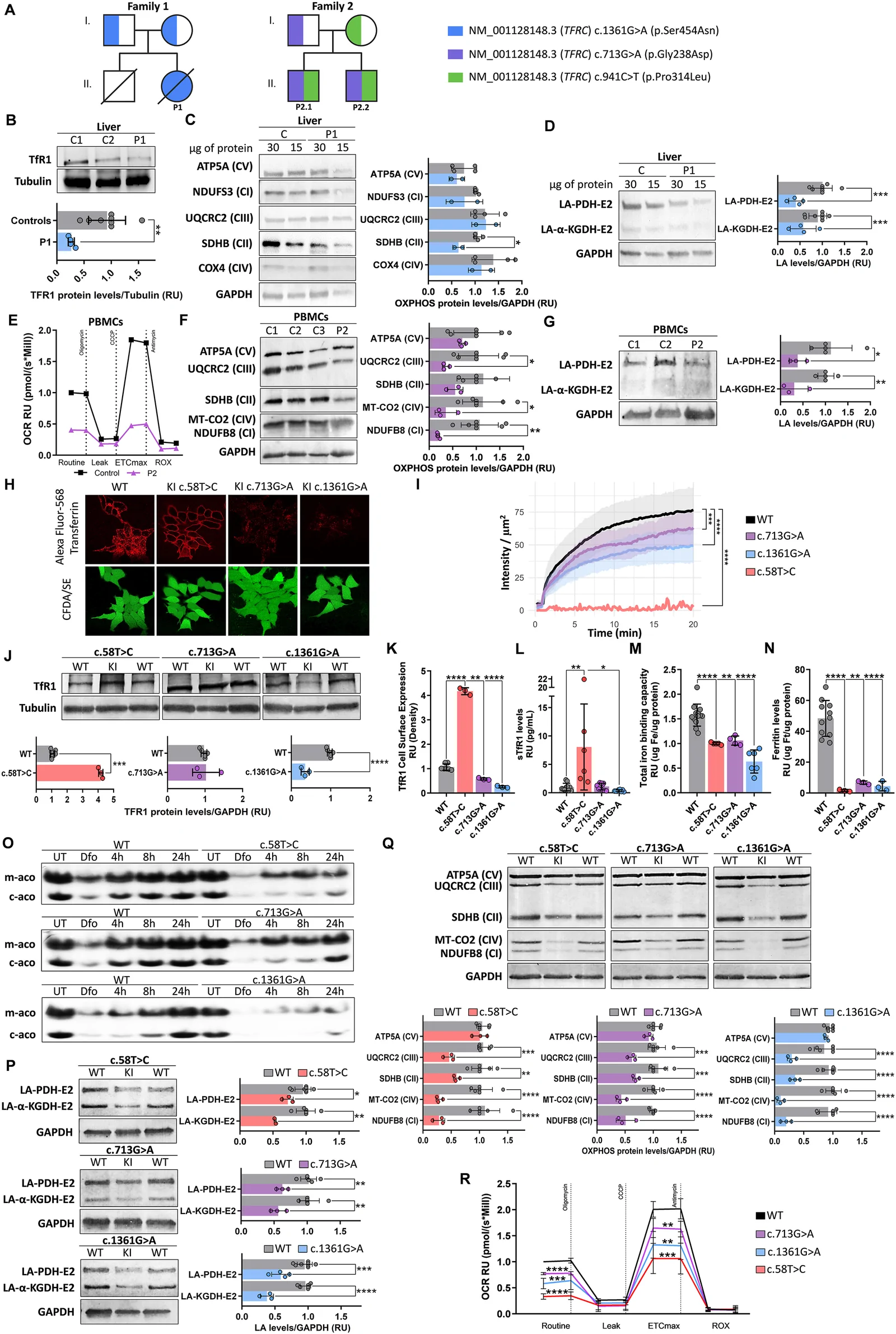
TFRC pedigree of both families and analysis of TfR1 and mitochondrial function in patient samples and in *TFRC* KI cellular models. **(A)** Family pedigrees showing three affected individuals carrying variants in *TFRC*. **(B)** Significant decrease of steady-state levels of TfR1 in the liver of P1. **(C)** Western blot analysis of representative subunits of the OXPHOS system in the liver of P1. Quantitative data showed a decrease of all complexes, except complex III. **(D)** Western blot analysis of lipoylated proteins (LA-PDH-E2 and LA-α-KGDH-E2) showed a significant deficiency in the liver of P1. **(E)** Analysis of mitochondrial respiration in fresh complete PBMCs from P2.1 resulted in an important reduction of both basal and ETCmax respiratory capacity. Oxygen Consumption Rate (OCR) was measured as pmol/(second × Millions of cells). Only one biological replicate was performed due to the lack of additional fresh material. Data are expressed as relative units (RU) to the control cells. **(F)** Western blot analysis of representative subunits of the OXPHOS system in complete PBMCs of P2.1 exhibited reduced steady-state levels of complexes I-IV. **(G)** Defective lipoylated proteins in complete PBMCs of P2.1 were also detected. **(H)** The Tf endocytosis rate was visualized using live cell imaging and a fluorescently red-labeled-Tf internalization assay. Representative confocal microscopy images of *in vitro* Alexa Fluor-568-labeled transferrin (Tf) uptake assay in HAP1 cell models. The c.58T>C KI model showed increased accumulation of Tf labelling on the cell membrane and the absence of intracellular endocytic vesicles. The c.713G>A and c.1361G>A models displayed reduced levels of both membrane-bound and endocytic Tf-labelling, revealing a significant delay of Tf internalization. Images were captured 15 min after adding labelled Tf (red) to the culture medium using a Zeiss LSM 5 live confocal microscope with 63x oil immersion objective. CFDA/SE staining (green) shows the cytosolic signal and indicates cell shape. The scale bars are 10 μm in length. **(I)** Quantification of Alexa Fluor-568-labeled Tf uptake. The results for TFRC KI cell models with the c.713G>A and c.1361G>A variants showed a significant delay of Tf uptake, on the basis of the reduced number of endosomes containing labeled Tf, while the uptake was absent for the cells harboring the c.58T>C variant. Images were acquired at intervals of 10 s over 20 min (120 time-frames). Fluorescence was quantified over at least two independent experiments. The number of total quantified cells per condition can be found in the Supplemental Methods section. The fluorescence intensities were normalized by the total cell area of each cell. Data are expressed as the normalized mean intensity (solid line) ± SD (shaded ribbons) for each comparison. **(J)** Western blot analysis of TfR1 in KI models revealed markedly elevated steady-state protein levels in the c.58T>C KI. In contrast, TfR1 levels in the c.713G>A KI were comparable to those in wild-type (WT) cells, while the c.1361G>A KI showed a significant reduction. **(K)** Flow cytometry analysis of cell surface TfR1 expression revealed that the c.58T>C KI model exhibited a fourfold increase in TfR1 levels, whereas the c.713G>A and c.1361G>A KI models showed reductions of approximately 40% and 70%, respectively. Three biological replicates were performed for each cell line. Values are given as relative units (RU) to the control cells. **(L)** Soluble TfR1 (sTfR1) levels in the culture medium showed elevated steady-state levels in the c.58T>C KI, while levels were comparable to WT cells in the c.713G>A KI, and were significantly decreased in the c.1361G>A KI. Between six and nine biological replicates for each cell line were studied. Values are given as relative units (RU) to the control cells. **(M)** Total iron-binding capacity analysis performed in cell homogenates showed a significant reduction in the three KI models compared to WT cells. The results were normalized for protein content and expressed as μg Fe/μg protein. **(N)** Ferritin (Ft) analysis performed in cell homogenates showed a significant reduction in the three KI models compared to WT cells. The results were normalized for protein content and expressed as μg Ft/μg protein. **(O)** All KI models showed defective Fe–S biogenesis, as evidenced by delayed recovery of both mitochondrial (m)- and cytosolic (c)-aconitase activities upon iron deprivation, and deferoxamine (Dfo) treatment, indicating impaired Fe–S reassembly and dysregulated biosynthesis of this cofactor. Three biological replicates were studied for each condition. **(P)** Western blot analysis of lipoylated PDH and α-KGDH E2 subunits showed a significant reduction of protein-bound lipoic acid (LA) in the three TFRC KI models. **(Q)** Western blot analysis of representative subunits of the OXPHOS system in the three TFRC KI cell models showed reduced steady-state levels of the complexes I-IV subunits, but the complex V subunit remained unchanged. **(R)** Analysis of mitochondrial respiration resulted in a significant reduction of both basal and maximal (ETCmax) respiratory capacity in the three TFRC KI cell lines. GAPDH was used as a loading control in all sections, except in section A, where alpha(α)-tubulin served as the loading control. PBMCs, peripheral blood mononuclear cells; OCR, oxygen consumption rate; ROUTINE, basal state; LEAK, residual oxygen consumption after oligomycin treatment; ETCmax, maximum oxygen consumption induced by carbonyl cyanide 3-chlorophenylhydrazone (CCCP) titration; ROX, residual oxygen consumption after antimycin A treatment, WT, wild-type; KI, knock-in. Quantitative data are given as the mean ± SD. *P*-values were calculated by two-tailed Student's *t*-test. ∗*p* < 0.05, ∗∗*p* < 0.01, ∗∗∗*p* < 0.001, ∗∗∗∗*p* < 0.0001. In each graph dots indicate the number of replicates.

The patient of family 1 (P1) experienced a fatal outcome and died 2.5 h after birth due to cardiac shock as a result of hypertrophic cardiomyopathy. In contrast, the two siblings of family 2 (P2.1 and P2.2), currently 10 and 4 years of age, are alive, but both suffer from severe microcytic anemia (Tables S2 and S3) and progressive neurological symptoms (Supplementary material).

The essential role of iron in cardiomyocytes and particularly, the report of mice lacking TfR1 in the heart^.^ exhibiting cardiac hypertrophy, cardiomegaly, and reduced activities of OXPHOS complexes^2^ led us to speculate that the homozygous *TFRC* variant (c.1361G>A; p.Ser454Asn) identified in P1 (Fig. 1A) may underlie the observed disease phenotype. Consistently, mitochondrial studies in P1 liver necropsy revealed low TfR1 levels, reduced steady-state levels of OXPHOS proteins and impaired protein lipoylation, indicating a generalized defect of mitochondrial energy metabolism and disrupted Fe–S cluster biogenesis (Fig. 1B–D). Concerning family 2, exome sequencing identified two missense *TFRC* variants in trans in both siblings (Fig. 1A; Fig. S1A). In this regard, despite the embryonic lethality of germline *Tfrc* knock-out mice,^1^ animal models with disruption of a single *Tfrc* allele are viable, although they develop microcytic anemia, like the siblings of this family. Despite normal or near-normal serum Ferritin (Ft) and Transferrin (Tf) levels (Tables S2 and S3), which are key indicators of iron deficiency, our patients presented anemia as a result of impaired endocytosis of the Fe-Tf/TfR1 complex, rather than iron deficiency, accounting for their unresponsiveness to iron therapy. In contrast to patients with CID, our patients showed only a slight decrease of IgG (Table S4) and normal lymphocyte profile (Table S5). Accordingly, these patients never had severe infections. P2.1 complete PBMCs exhibited severe impairment of mitochondrial respiratory capacity, reduced steady-state levels of OXPHOS proteins and defective protein lipoylation (Fig. 1E–G), closely resembling the alterations observed in P1 liver tissue. These findings suggest that patients of both families are likely to suffer from the same disease, albeit with variable severity and distinct clinical presentations. Mitochondrial function and lipoic acid biosynthesis have not been studied in CID patients or in the CID mouse model,^3^ although they are likely to have similar implications to our patients. Notably, Aba et al in 2024 noticed significantly impaired oxidative capacity in total lymphocytes of his CID patient, pointing to mitochondrial impairment.^5^ Our findings provide the first evidence that *TFRC* disease-associated variants impair mitochondrial function and protein lipoylation in human tissues.

The need to demonstrate the pathogenicity of the novel *TFRC* variants prompted us to perform a more comprehensive functional characterization. However, due to the lack of patient-derived cell lines, we generated CRISPR/Cas9 *TFRC* knock-in (KI) cell models for the reported c.58T>C mutation,^3^, ^4^, ^5^ and for the newly identified variants except for the c.941C>T variant (Table S6 footnote). Our aim was to carry out a comparative study among these variants, representative of the three different phenotypes: CID, microcytic anemia and hypertrophic cardiomyopathy. We first investigated the Tf internalization rate by tracking red-labeled Tf throughout its endocytic cycle. Consistent with previous studies, the c.58T>C KI model exhibited increased levels of surface-bound Tf, indicating an over-accumulation of the Tf/TfR1 complex at the plasma membrane and a complete absence of labeled Tf inside the cell, suggesting a total loss of Tf endocytosis (Fig. 1H and I). Therefore, our HAP1 c.58T>C KI successfully recapitulates the model previously described in fibroblasts of CID patients.^3^ In contrast, the KI models carrying the newly identified c.713G>A and c.1361G>A variants displayed a marked delay in Tf uptake, as evidenced by the reduced number of Tf-containing endosomes compared to wild-type (WT) cells (Fig. 1H and I). Unlike the c.58T>C KI, cells harboring the c.713G>A and c.1361G>A variants retained the ability to bind to and internalize Tf but at a significantly reduced rate (Fig. 1I). On the other hand, the KI c.58T>C cells showed high levels of surface and soluble TfR1. Conversely, the KI c.713G>A cells displayed TfR1 levels comparable to those of WT cells, while the KI c.1361G>A cells showed a significant reduction in TfR1 levels (Fig. 1J–L) Interestingly, the lowest levels of TfR1 corresponded to the KI variant c.1361G>A, which also presented the lowest TfR1 protein expression and was associated with the lethal cardiomyopathic phenotype. The total Fe-Tf-binding capacity was moderately reduced in all three KI cells (Fig. 1M) whereas Ft levels were extremely low in all KI models (Fig. 1N). These findings are consistent with defective Fe–S cluster biogenesis, as further evidenced by the altered dynamics of Fe–S cluster disassembly and reassembly (Fig. 1O). Notably, this defect appears again to be more pronounced in the cardiomyopathy-associated genotype. In addition, defective protein lipoylation (Fig. 1P) along with an overall defect of the mitochondrial function (Fig. 1Q and R), and low enzymatic activities of complexes I–IV (Fig. S2) closely recapitulated the alterations detected in patients’ tissues. Altogether, our results provide evidence of impaired TfR1 function, and consequently, the identified variants were reclassified as likely pathogenic (Table S1).

Interestingly, despite the critical role of TfR1 in erythropoiesis, some CID patients had only mild anemia, while others did not show anemia at all, but all of them presented hypogammaglobulinemia.^3^, ^4^, ^5^ Remarkably, the two TFRC mutations identified in patients with CID disrupt the TfR1 internalization motif, leading to the complete absence of Fe-Tf/TFR1 endocytosis.^3^, ^4^, ^5^ Jabara et al^3^ demonstrated a compensatory effect of STEAP3 in erythroblasts of these patients, suggesting that this mechanism may protect CID patients from developing severe anemia but not from immunodeficiency, as STEAP3 was poorly expressed in T and B cells.^3^ The *TFRC* variants in our patients are not located in the internalization motif but in the apical domain (microcytic phenotype) or in the protease-like domain (cardiomyopathic phenotype). It might be that the compensatory effect mediated by STEAP3 operates effectively only in the context of entirely absent Tf endocytosis, but not when the uptake is merely delayed. In such a scenario, it can be speculated that once inside the cell, the Fe-Tf/TfR1 complex can function normally in CID patients, as the mutation affects only the internalization motif, and hemoglobin can be produced in erythroblasts, while this process may not proceed efficiently in patients with variants that target other TfR1 motifs, leading to reduced hemoglobin production, as it happens in family 2 patients. Regarding P1, with hypertrophic cardiomyopathy, STEAP3 does not appear to confer protection, likely due to its low expression in cardiac muscle (Fig. S3) or to another yet unknown factor.

Due to the fundamental role of TfR1 in iron homeostasis and cell survival, we investigated whether cell viability could be compromised. Indeed, all KI cell lines showed increased apoptosis, especially under conditions of low iron availability. Notably, the extent of cell death correlated with patients’ clinical severity, being higher in the cells harboring the c.1361G>A variant (Fig. S4A–S4C). Interestingly, we observed that mitochondrial dysfunction and cell death were partially attenuated by nicotinamide riboside supplementation (Fig. S4D–S4F), suggesting a potential therapeutic benefit for patients with TfR1 deficiency, consistent with findings previously reported in mice.^2^

Despite some limitations, such as the limited availability of patients’ samples or the inability to generate one KI model, this study demonstrates for the first time that disease-associated *TFRC* variants cause severe mitochondrial dysfunction by disrupting Tf uptake and iron homeostasis, resulting in OXPHOS deficiency, altered Fe–S cluster biogenesis, defective protein lipoylation, reduced cellular respiration, and a variable degree of apoptotic cell death. Therefore, our findings broaden the phenotypic and functional spectrum of TfR1 deficiency and suggest the likelihood of additional patients beyond CID.

## CRediT authorship contribution statement

**Gerard Muñoz-Pujol:** Writing – original draft, Visualization, Software, Project administration, Investigation, Formal analysis, Data curation. **Elise J. Huisman:** Writing – original draft, Visualization, Validation, Supervision, Investigation, Formal analysis, Data curation, Conceptualization. **Juan-Francisco Mesa:** Writing – review & editing, Validation, Investigation, Formal analysis, Data curation. **Marieke Joosten:** Writing – original draft, Visualization, Validation, Methodology, Investigation, Formal analysis, Data curation. **Annick Devos:** Writing – review & editing, Methodology, Formal analysis, Data curation. **Miguel Fernandez-Burriel:** Writing – original draft, Visualization, Validation, Investigation, Formal analysis, Data curation. **Olatz Ugarteburu:** Writing – original draft, Visualization, Methodology, Investigation, Formal analysis, Data curation. **Eulàlia Segur-Bailach:** Writing – review & editing, Writing – original draft, Visualization, Methodology, Investigation, Data curation. **Sabrina Gea-Sorlí:** Writing – review & editing, Writing – original draft, Methodology, Investigation, Formal analysis, Data curation. **Sonia Moliner:** Writing – review & editing, Visualization, Validation, Methodology, Formal analysis. **Laia Farré-Tarrats:** Writing – review & editing, Methodology, Investigation, Formal analysis, Data curation. **Mariona Guitart-Mampel:** Writing – review & editing, Validation, Methodology, Investigation, Formal analysis, Data curation. **Gianluca Arauz-Garofalo:** Writing – review & editing, Writing – original draft, Supervision, Software, Methodology, Formal analysis, Data curation. **Marina Gay:** Writing – review & editing, Validation, Software, Methodology, Investigation, Formal analysis. **Neus Villamor:** Writing – review & editing, Validation, Methodology, Formal analysis, Data curation. **Gemma Martín:** Writing – review & editing, Validation, Methodology, Formal analysis, Data curation. **María Calvo:** Writing – review & editing, Writing – original draft, Supervision, Software, Methodology, Data curation. **Gloria Garrabou:** Writing – review & editing, Writing – original draft, Supervision, Investigation, Data curation. **María J. Macias:** Writing – review & editing, Writing – original draft, Visualization, Software, Methodology, Data curation. **Laura Gort:** Writing – review & editing, Visualization, Methodology, Investigation, Formal analysis, Data curation. **Judit García-Villoria:** Writing – review & editing, Validation, Methodology, Investigation, Data curation. **Cristina Fillat:** Writing – review & editing, Writing – original draft, Validation, Supervision, Investigation, Data curation. **Frederic Tort:** Writing – review & editing, Writing – original draft, Visualization, Validation, Supervision, Resources, Investigation, Funding acquisition, Data curation, Conceptualization. **Antonia Ribes:** Writing – review & editing, Visualization, Validation, Supervision, Resources, Project administration, Investigation, Funding acquisition, Data curation, Conceptualization.

## Ethics declaration

Informed consent was obtained from the families involved in the study, and the procedures related to primary human samples were approved by the Research Ethics Committee of Hospital Clinic of Barcelona (Reference number: HCB/2021/0006).

## Data availability

All data reported in this manuscript is available from the corresponding authors upon request.

## Funding

This research was supported by the projects PI19/01310 and PI22/00856, funded by the Instituto de Salud Carlos III (ISCIII) and co-funded by the 10.13039/501100000780European Union. This study was supported by the Centro de Investigación Biomédica en Red de Enfermedades Raras (CIBERER). CIBERER is an initiative of the Instituto de Salud Carlos III (Ministerio de Ciencia e Innovación, Spain). M.G.-M. was supported by CD21/00019 ISCIII-FSE+. This study was also supported by the Agència de Gestió d’Ajuts Universitaris i de Recerca (AGAUR) (2021:SGR 01423 and SGR00866) and the 10.13039/100015439CERCA Programme/10.13039/501100002809Generalitat de Catalunya. MJM is an ICREA Program Investigator.

## Conflict of interests

The authors declare no competing interests.
