# Supplementary material for: TfR1 deficiency beyond combined immunodeficiency. Pivotal role of mitochondrial function and iron-sulfur cluster biogenesis in disease pathogenesis
Source: Genes Dis. 2026 Feb 19;14(1):102094. doi: 10.1016/j.gendis.2026.102094 (PMC13562382; doi:10.1016/j.gendis.2026.102094)
Supplement: Supplementary file 1 [file mmc1.docx]

**SUPPLEMENTARY MATERIAL**

**Inventory of Supplementary Information**

**Supplementary Material and Methods**

**Subjects**

**Methods**

**Supplementary Figure S1.** TFRC variant validation by Sanger sequencing of both families, cerebral MRI images of family 2 patients and tridimensional structure of TfR1 protein

**Supplementary Figure S2.** Enzymatic activity of mitochondrial respiratory chain complexes I-IV in *TFRC* KI cell lines

**Supplementary Figure S3.** *STEAP3* RNA expression across human tissues (GTEx**).**

**Supplementary Figure S4.** Impaired apoptosis and partial improvement with Nicotinamide Riboside supplementation in *TFRC* KI cellular models.

**Supplementary Table S1.** Classification of variants according to the criteria of the American College of Medical Genetics and Genomics, and the Association for Molecular Pathology (ACMG/AMP).

**Supplementary Table S2**. Patient’s (P2.1 and P2.2) hemogram and iron studies.

**Supplementary Table S3**. Patient’s (P2.1 and P2.2) hemogram and iron studies, following chronic blood transfusion.

**Supplementary Table S4.** Patients’ (P2.1 and P2.2) immunoglobulin levels in serum.

**Supplementary Table S5.** Lymphocyte **immunophenotypic profile** of P2.1 and P2.2

**Supplementary Table S6**. crRNAs, ssODNs and primers used in this study.

**Supplementary References**

**Supplementary Material and Methods:**

**Subjects**

**Patient 1 (P1)** was the second daughter of non-consanguineous parents (Family 1). Her older brother died suddenly at home seven days after birth, autopsy or other post-mortem studies were not performed. Notably, the mother exhibited a lifelong history of borderline hemoglobin (Hb) levels and reduced mean corpuscular volume (MCV). No other remarkable clinical data were detected. Pregnancy was uneventful, and fetal ultrasound scans showed no signs of pathology. Apgar score was 9/10, and no postpartum resuscitation was required. Birth weight, length, and head circumference were normal. The patient was admitted to the neonatal unit waiting for the mother's discharge. Her vital signs were normal, but at two and a half hours of life, she became unresponsive to stimuli, exhibited poor coloring, and lacked detectable heartbeat or pulse. Cardiopulmonary resuscitation was initiated, involving intubation, umbilical vein cannulation, and administration of intratracheal and intravenous adrenaline. Despite administering repeated doses of adrenalin and cardiopulmonary resuscitation efforts for 45 minutes, the patient unfortunately died. A postmortem blood sample was obtained. Results from routine biochemical analysis showed only abnormally altered AST 233 U/L (reference values: 28-98) and generalized hyperaminoacidemia. No additional sample was available to perform a complete blood count.

At autopsy, a markedly enlarged heart was observed, extending well beyond the lower pulmonary margin. Significant hypertrophy was noted in both ventricles, accompanied by dilation of both atria. The presence of cells with fatty appearance in cardiac muscle, liver, and kidney was suggestive of an inherited metabolic disorder. Periodic Acid–Schiff (PAS) staining was negative. Unfortunately, liver tissue was the only well-preserved sample available for further analyses.

WES identified a homozygous *TFRC* missense variant, c.1361G>A (p.Ser454Asn) in the index case (P1) of famil1. Sanger sequencing validated the homozigosity of P1 and parents’ segregation (Figure S1A). The variant was classified as Variant of Uncertain Significance (VUS) (Table S1)

**Patient 2.1 (P2.1)** is the first son of non-consanguineous parents (Family 2). Family background was unremarkable, except for the presence of microcytic erythrocytes in the mother, but without anemia. P2.1 was born prematurely at 36 1/7 weeks of gestational age due to maternal indications. Birth weight and head circumference at birth was in the 10th percentile. He was admitted to the pediatric intensive care unit at the age of 6 months due to increasing feeding problems and rapidly growing of head circumference above the 90th percentile. Computed tomography scan revealed multiple epidural and subdural frontoparietal bleedings/hygromas with ventricular involvement. Anemia was observed upon admission, showing very low levels of Hb (4 g/dL; reference range:12-13) and MCV (42 fL; reference range:78-83) with normal neutrophil and platelet counts. Iron deficiency was suspected but he showed no response to oral iron administration. Hygromas and reduced Factor IX (FIX), around 30%, led to the suspicion of hemophilia B but no mutations in F9 were found, and at the age of one year FIX levels normalized. Normal plasma and urine metabolic studies discarded the most frequent inherited metabolic disorders. Neurological examination follow-up showed nystagmus, and progressive spastic diplegia in the lower extremities. Magnetic Resonance Imaging (MRI) of the brain showed hygromas as well as abnormal white matter signals (Figure S1B-S1E). MRI of the spine did not explain the diplegia, but on repeat MRI two years later, an arteriovenous malformation was discovered, causing local compression around L5/S1. Follow-up of the patient was lost for two years. Upon return, examination showed normal cognitive development, but Hb remained low (baseline Hb levels as low as 5 g/dL) with extremely reduced MCV, ranging from 45 to 50 fL and reticulopenia. These results led us to the diagnosis of congenital anemia with microcytic characteristics, that together with the clinically evident fatigue prompted the initiation of treatment, starting with two administrations of intravenous Venofer®. However, due to the lack of benefit, erythrocyte transfusion was performed, resulting in improved Hb levels. Serum total iron-binding capacity and Tf remained slightly low, while iron, ferritin (Ft) and Tf saturation were normal (Table S2). Additional iron studies, including hepcidin (a hepatic hormone essential for maintaining iron homeostasis), and protoporphyrins (critical for heme production), were within the reference levels (Table S3).

WES of this family showed, two in trans missense variants -c.713G>A (p.Gly238Asp) and c.941C>T (p.Pro314Leu) in both siblings. Segregation analysis by Sanger sequencing validated the variants and confirmed that each parent was heterozygous for the corresponding variant, the father carried the c.713G>A variant and the mother carried the c.941C>T variant. (Figure S1A). Both variants were classified as VUS (Table S1). Following the molecular diagnosis, soluble TfR1 levels were measured and found to be low (42 µg/dL; reference range 83-176). At eight years of age, immunoglobulin levels were normal, while a recent assessment at 10 and at 10 years 9 months of age revealed a slight deficiency of IgG levels at both ages (Table S4). He also required cataract surgery twice.

**Patient 2.2 (P2.2)** is the younger brother of Patient 2.1. Pregnancy and delivery were uneventful. The neonatal period was normal. However, at one month of age, he was screened for congenital anemia, which revealed microcytic anemia (Hb 6.9 g/dL, MCV 57 fL) normal neutrophil and platelet counts, like his brother. Genetic study demonstrated the same genotype of his brother (P2.1).Additional iron metabolism parameters are provided in Table S2-S3.Immunoglobulin levels performed at 4 years of age and recently, at 4 years 9 months of age showed a slight deficiency of IgG at both ages (Table S4). Brain MRI performed at 1 year of age revealed abnormal white matter development with degenerative changes in the pons. Follow-up MRI imaging at 2 years of age showed, progression of these abnormalities (Figure S1F-S1H). At the same age, due to the gradual onset of diplegia, a thoracic MRI was conducted, revealing novel occurrence of arterio-vascular malformations in the thoracic spinal canal. Nystagmus was also noted starting at 11 months of age. Echocardiogram was completely normal.

**Methods**

**Structural analysis of TFR1 protein.**

*In silico* structural analysis indicates that p.Ser454Asn substitution is compatible with the canonical TfR1 3D structure of the protein, as the Asn side chain can still interact with three nearby residues, Asp410, Asp160 and Arg 409 (Fig. S1I and S1J). However, changes at positions Gly238 and Pro314 are predicted to have a destabilizing effect on the protein structure. In the case of the p.Gly238Asp substitution, the presence of the Asp side chain reduces the space available for Lys267, whose side chain is displaced from its original position. In the case of Pro314Leu substitution, although the side chains are of similar size, the substitution increases the freedom of the backbone in the loop. The new Leu residue at this position allows for additional backbone orientations compared to the more rigid Pro (Fig. S1J)

**HAP1 cell culture.**

HAP1 cell culture HAP1 parental cell line (C631) was purchased from Horizon Discovery Ltd (Waterbeach, UK). HAP1 is a human near-haploid cell line derived from KBM-7 cells, a chronic myelogenous leukemia cell line. HAP1 cells were grown in Iscove’s Modified Dulbecco’s Medium (IMDM; 16SH30228-01, GIBCO, Waltham, MA USA), and supplemented with 10% fetal bovine serum (FBS) and 1% Penicillin/Streptomycin. Cells were grown in 25 cm2 flasks at 37°C, and 5% carbon dioxide (CO2). When indicated, HAP1 KI model cells were treated with 80 μM nicotinamide riboside chloride (NR, SMB00907, Sigma-Aldrich, St Louis, MO, USA) for 48h / 72 h or with 50 mM deferoxamine (Dfo) for 18 h. HAP1 cells typically diploidize spontaneously over time^1^. Thus, before CRISPR/Cas9 nucleofection experiment, early passage of HAP1 cells were sorted to obtain a haploid-enriched population^2^. Fluorescence activated cell sorting (FACS) was performed staining cells for DNA content with Hoechst 33342 (62249, Thermo Fisher Scientific, Waltham, MA USA) at 10 μg/mL of complete IMDM for 30 min at 37°C. The isolated haploid cells were expanded before CRISPR/Cas9 nucleofection.

**Generation of *TFRC* knock-in cell line models using CRISPR/Cas9.**

To demonstrate the pathogenic effect of the identified variants and to investigate their functional impact, we generated CRISPR/Cas9-mediated KI models using the human near haploid cell line HAP1^3^. The advantage of HAP1 cells is that, since they are haploid, the CRISPR/Cas9-mediated gene editing only needs to modify one allele to expose the mutation-associated phenotype. All CRISPR/Cas9 reagents were designed and purchased from Integrated DNA Technologies (IDT, Coralville, IA, USA). Delivery procedures and off-target in silico analysis were conducted following IDT recommendations (https://eu.idtdna.com/). A complete list of all CRISPR/Cas9 reagents used in this work can be found in Supplementary Table S6. Each *TFRC* modeled variant needed the customization of a CRISPR RNA (crRNA) targeting the genomic region close to the nucleotide of interest and a single-stranded oligonucleotide (ssODN) repair template containing the desired variant (Ultramer DNA Oligos, IDT). CRISPR/Cas9 genetic edition was performed as described^3^. Briefly, before nucleofection, equimolar concentrations of each Alt-R crRNA and Alt-R trans-activating crRNA (tracrRNA) oligos were used to prepare crRNA:tracrRNA duplexes (sgRNA). Oligos were annealed by heating at 95°C for 5 min in a heating block and the mix was slowly cooled to room temperature. For each nucleofection reaction, ribonucleoprotein (RNP) was prepared combining the specific pre-duplexed sgRNA and Alt-R Streptococcus pyogenes Cas9 Nuclease V3 to a final Cas9:sgRNA concentration of 4:4.8 µM and incubated at room temperature for up to 20 min. Nucleofection was carried out using the Neon Transfection System (MPK5000, Thermo Fisher Scientific, Waltham, MA, USA). Haploid HAP1 cells were counted with a Scepter 2.0 Cell Counter (MERCK, Darmstadt, Germany) and 4x105 cells were resuspended in 9 µL of Resuspension Buffer R (MPK1096, Thermo Fisher Scientific, Waltham, MA, USA). In the same reaction tube, 1 µL of RNP and 1.2 µL of ssODN were added. 10 µL of cells with RNP complex mixture were nucleofected at 1,800 V, 10 ms pulse width, 3 pulses. After nucleofection, cells were seeded into 6-well plates containing prewarmed complete IMDM without antibiotics. Nucleofected cells were expanded, and limiting dilution of HAP1 pools was performed to obtain single-cell-derived clones. Validation of HAP1 clones was achieved by PCR and Sanger sequencing of the targeted *TFRC* genomic region using specific primers (available upon request). Clones with the desired *TFRC* variants in homozygosis were selected as knock-in isogenic cell lines and expanded for further experiments.

**Western blot Protein expression analysis.**

Muscle and liver from P1 and PBMCs from P2.1 were homogenized on ice in tissue extraction buffer (250 mM mannitol, 75 mM sucrose, 10 nM Tris-HCl, 0.1 mM EDTA) followed by centrifugation for 20 min at 650 g at 4ºC. HAP1 cells grown in 25 cm2 flasks were scrapped on ice with RIPA buffer (0.1% SDS, NP40 1%, Sodium deoxycholate 0.5% in PBS) containing protease inhibitors (cOmpleteTM, Mini, EDTA-free Protease Inhibitor Cocktail 4693159001, Roche, Indianapolis, IN, USA), and centrifuged for 10 min at 10.000 g at 4ºC. Protein concentrations of the cleared lysates were quantified using a Bradford protein assay kit (5000116, Bio-Rad, Hercules, CA). Protein extracts were mixed with 4x Loading buffer (50 mM Tris-HCl pH 6.8, 2% SDS, 10% glycerol, 1% β-mercaptoethanol, 12.5 mM EDTA, 0.02% bromophenol blue), heated for 10 min at 95ºC, and subjected to SDS-Polyacrylamide Gel Electrophoresis. Proteins were transferred to nitrocellulose membranes and immunostained with specific antibodies: TfR1 (13-6800, Invitrogene, Waltham, MA, USA), SDHA (MS204, MitoSciences, Eugene, OR, USA), ATP5A (ab14748, Abcam, UK), NDUFS3 (ab14711, Abcam, UK), UQCRC2 (ab14745, Abcam, UK), COX4 (LS‑B10751, Cell Signaling, Danvers, MA, USA), Mitococktail (45-8199, Invitrogene, Waltham, MA, USA), lipoic acid (437695, Calbiochem, Germany), Caspase-3 (9664S, Cell Signaling, Danvers, MA, USA), PARP-1 (sc-8007, Santa Cruz Biotechnology, Dallas, TX, USA), p62 (ab56416, Abcam, UK), LC3 (L7643, Sigma-Aldrich, St Louis, MO, USA), Tubulin (ab176560, Abcam, UK), and GAPDH (sc-47724, Santa Cruz Biotechnology, Dallas, TX, USA). Proteins were visualized by colorimetric detection (Opti-4CNTM Substrate Kit, Bio-Rad, Hercules, CA, USA). Images were analyzed and quantified with ImageJ software (Bethesda, MD, USA).

**Aconitase in-gel activity.**

HAP1 cell pellets were resuspended in 200 µL lysis buffer (25 mM Tris-HCl pH 8.0, 1% Triton X-100, 100 mM NaCl, 2 mM citrate, 0.5 mM PMSF, and 10% glycerol) and extracts were centrifuged at 10.000g, 10 minutes at 4ºC. Supernatants were mixed with Loading Buffer 5x (25 mM Tris-HCl pH 8.0, 10% glycerol, and 0.025% bromophenol blue) and electrophoresed in 8% acrylamide gels. The separating gels contained 132 mM Tris-base, 132 mM borate, 3.6 mM citrate, 1% APS, and 1% TEMED). The stacking gels were composed of 4% acrylamide, 67 mM Tris-base, 67 mM borate, 3.6 mM citrate, 1% APS, and 1% TEMED. Running buffer contained 25 mM Tris pH 8.3, 192 mM glycine, and 3.6 mM citrate. Electrophoresis was carried out at 180 V at 4ºC for 3 hours. Aconitase activity buffer consisted of 100 mM Tris pH 8.0, 1 mM NADP, 2.5 mM cis-aconitic acid, 5 mM MgCl2, 1.2 mM methylthiazolyl-diphenyl-tetrazolium bromide (MTT), 0.3 mM phenazine methosulfate, and 5 U/mL isocitrate dehydrogenase (Sigma-Aldrich, St. Louis, USA)^4,5^. Gels were incubated with activity buffer in the dark at 37ºC for 30 minutes. When indicated cells were treated with Dfo, as described above.

**In vitro Alexa Fluor 568-labeled Tf uptake assay.**

HAP1 cells were cultured on poly-D-lysine-coated (A3890401, GIBCO, Waltham, MA USA) 25-mm diameter-round cover glasses (72225-01, Electron Microscopy Sciences, Hatfield, PA) in complete IMDM and stained with 5-(and-6)-Carboxyfluorescein diacetate N-succinimidyl ester (CFDA/SE, V12883, Invitrogene, Waltham, MA, USA) 0,5 µM in PBS at 37ºC for 15 min and rinsed twice in serum-free IMDM for 30 min. This led to Tf starvation of 45 min. For imaging, cells were mounted in an Attofluor chamber adapted for perfusion (Thermo Fisher Scientific, Waltham, MA USA) and maintained in serum-free IMDM at 37°C and 5% CO_2_. Live cell imaging was performed in a Zeiss LSM880 laser scanning spectral confocal microscope (Carl Zeiss, Jena, Germany) equipped with an Axio Observer 7 inverted microscope, blue diode (405 nm), Argon (488 nm), diode pumped solid state (561 nm) and HeNe (633 nm) lasers, a Plan Apochromat 63x oil (NA 1.4) immersion objective lens and an incubation system with temperature (37ºC), CO_2_ (5%) and humidity control. CFDA/SE and AlexaFluor-568-conjugated Tf (Tf-568, T23365, Invitrogene, Waltham, MA, USA) images were acquired sequentially using a 488 nm and 561 laser lines and emission detection ranges 500-550 nm and 571-625 nm, respectively, and the confocal pinhole set at 1 Airy units. Images were acquired at an interval of 10 sec during 20 min (120 time frames). On the third timeframe, Tf-568 (5 μg/mL in serum-free IMDM) was injected to track endocytic TfR1:Tf-568 vesicles formation and endosome recycling. Fluorescence was quantified over at least two independent experiments.

Quantitative analysis of internalized Tf was performed using FIJI, a distribution of Image J (Wayne Rasband, NIH, USA). A custom-made macro was programmed with instructions for the automated image analysis pipeline and can be found at <https://github.com/UB-BioMedMicroscopy/Transferrin-Endocytosis/releases/tag/v1.0.1>. The following workflow was automated in an ImageJ macro script to analyze cell by cell, and frame by frame, for all the internalization experiments. First, to segment cells frame by frame, CFDA-SE channel images were filtered. To specifically quantify internalized Tf the intensity and number of Tf-loaded endosomes were quantified from the cytoplasm in each cell in each time frame. Plasma membrane intensity was obtained by subtracting the cytoplasm cell region from the total cell region. Numbers of total quantified cells per condition: WT (N=115); KI c.58T>C (N=40); KI c.713G>A (N=40); KI c.1361G>A (N=25). The fluorescence intensities were normalized per cell by its total cell area in every image. Welch two sample *t*-test was used for comparison between time-series: WT vs c.58T>C, WT vs c.713G>A, and WT vs c.1361G>A.

**Supplementary Figure S1. TFRC variant validation by Sanger sequencing of both families, cerebral MRI images of family 2 patients and tridimensional structure of TfR1 protein. A**) Sanger sequencing validation and parents segregation of family 1 and Sanger sequencing of family 2 parents showing also segregation of the identified mutation. **B-C**) Cerebral MRI images of P2.1at 3years of age showed widened supratentorial and infratentorial ventricle systems and widened peripheral liquor spaces. Liquor is heterogeneous in signal and slightly higher in signal on T1 images. No signs of ischemic zones. **D-E**) normal pons. **F-G**) Cerebral images of P2.2 at the age of 2 years showed a cruciform bright T2- signal in the pons (hot cross bun sign) with diffusion restriction. The sagittal T1-image (**H**) showed an abnormal low signal in the pons. **I**) Cartoon model of the native wild-type TfR1 dimer structure. One monomer is color-coded in yellow and the other one in orange. Circles highlight the regions containing the variants of interest in each monomer. For clarity, variants are depicted in both monomers. Altered residues are marked in dark blue. In the case of Gly238, which lacks a side chain, we have only indicated the backbone. **J**) Structural analysis of the newly identified variants in the *TFRC* gene: c.713G>A (p.Gly238Asp), c.941C>T (p.Pro314Leu), and c.1361G>A (p.Ser454Asn). The steric effects of changing Gly238 to Asp are illustrated by showing all Asp rotamers, revealing the lack of space to accommodate the Asp side chain at the Gly site. Changes observed at position 238 (Gly to Asp) and 314 (Pro to Leu) are likely to have a destabilizing impact in the tridimensional structure of the protein, whereas the Ser454Asn change appears to be compatible with the structure.

**Supplementary Figure S2. Enzymatic activity of mitochondrial respiratory chain complexes I-IV in *TFRC* KI cell lines**. Enzymatic activity of mitochondrial respiratory chain complexes I-IV showed a marked decrease in all three *TFRC* KI cell lines compared to WT cells. Activities were normalized for protein content and for citrate synthase (CS) activity (nmol/min × mg protein × U CS). Data are given as relative units (RU) to the control cells and as the mean ± SD; p-values were calculated by two-tailed Student’s t-test *p<0.05, **p<0.01, ***p<0.0001, ***p<0.00001.

**Supplementary Figure S3. *STEAP3* RNA expression across human tissues (GTEx).** Bar plot showing normalized transcript expression (nTPM) of *STEAP3* across human tissues based on the GTEx dataset, as presented in the Human Protein Atlas (<https://www.proteinatlas.org/>; consulted on 12 December 2025). The arrow indicates *STEAP3* RNA expression in heart muscle.

**Supplementary Figure S4. Impaired apoptosis and partial improvement with Nicotinamide Riboside supplementation in *TFRC* KI cellular models.** A) Negative widefield images from representative DAPI-stained nuclei. Nuclear fragmentation events are indicated with an arrow. The scale bars are 10 μm in length. B) Bar plot showing the percentage of fragmented nuclei in the absence and in the presence of the iron chelator Deferoxamide (Dfo) in the culture medium. Results showed higher amounts of fragmented nuclei in the KI cells, Dfo (-), compared to WT cells. However, differences increased significantly upon Dfo addition, Dfo (+). Three biological replicates were performed per each condition. C) Western blot analysis of PARP1 in response to iron chelation after 18 hours of treatment with 10 μM or 50 μM Dfo; 50 μM Dfo treatment increased the amounts of cleaved PARP1 in the KI cell lines, consistent with apoptosis activation. GAPDH was used as loading control. D) Percentage of fragmented nuclei in untreated (UT) and 80 μM nicotinamide riboside (NR)-treated cells for 48 h and 72 h, respectively. A gradual decrease of fragmented nuclei in response to NR treatment was observed. Upon 72 h of NR treatment, c.58T>C and c.1361G>A KI models showed a significant reduced percentage of fragmented nuclei compared to their corresponding UT KI cells. In each graph dots indicate the number of replicates. E) Upon 48h of NR treatment mitochondrial respiration significantly improved in terms of basal and maximal respiratory capacity in all KI cell models. F) Upon 72h of NR treatment the mitochondrial respiration resulted in slightly higher improvement than that seen at 48h. Two biological replicates were performed for each condition in both E and F sections. OCR was measured as pmol/second × millions of cells. UT, untreated; NR, nicotinamide riboside; WT, wild-type; KI, knock-in; OCR, oxygen consumption rate; ROUTINE, basal state; LEAK, residual oxygen consumption after oligomycin treatment; ETCmax, maximum oxygen consumption induced by carbonyl cyanide 3-chlorophenylhydrazone (CCCP) titration; ROX, residual oxygen consumption after antimycin A treatment. Data are given as relative units (RU) to the control cells and as the mean ± SD; p-values were calculated by two-tailed Student’s t-test *p<0.05, **p<0.01, ***p<0.0001.

**Supplementary Table S1.** Classification of variants according to the criteria of the American College of Medical Genetics and Genomics, and the Association for Molecular Pathology (ACMG/AMP).

| **Variant** | **ACMG/AMP Classification** | **ACMG/AMP classification after functional studies** |
| --- | --- | --- |
| **c.713G>A** | PM2 (VUS) | PS3 (Likely pathogenic) |
| **c.941C>T** | PM2, PP3 (VUS) | PS3 (Likely pathogenic)^*^ |
| **c.1361G>A** | PM2 (VUS) | PS3 (Likely pathogenic) |

VUS, variant of uncertain significance; PM2, pathogenic moderate; PP3, pathogenic supporting 2; PS3, pathogenic strong 3 (well-established functional studies showing damaging effect on the gene or gene product). *The pathogenicity of the c.941C>T variant has been reported in an abstract^6^, in which transferrin (Tf) internalization was impaired in a patient homozygous for this variant, supporting its reclassification as PS3.

**Supplementary Table S2**. Patient’s (P2.1 and P2.2) hemogram and iron studies**.**

| **Patient** | **P2.1** | **Age-matched reference values** | **P2.2** | **Age-matched reference values** |
| --- | --- | --- | --- | --- |
| **Age**  **(On transfusión)** | **8 years**  **(Yes)** |  | **3 months**  **(No)** |  |
| Hb (g/dL) | **9.8** | 10-15 | **6.9** | 9.4-13 |
| MCV (fL) | **53** | 76-97 | **59** | 84-106 |
| Reticulocyte count (x10E9/L) | **40** | 42-70 | 47 | 50-80 |
| Total Iron binding capacity (TICB) (µg/dL) | **212** | 250-420 | Not done |  |
| Transferrin saturation (%) | 50 | 20-45 | 46 | 20-45 |
| Iron (µg/dL) | 11 | 10-30 | 15 | 10-30 |
| Ferritin (µg/L) | 194 | 10-320 | **390** | 10 - 375 |
| Transferrin (mg/dL) | **150** | 203-360 | **130** | 215 - 380 |

Altered values are outlined in bold.

**Supplementary Table S3.** Patient’s (P2.1 and P2.2) hemogram and iron studies, both following chronic blood transfusion.

| **Patient** | **P2.1** | **Age**  **matched reference values** | **P2.2** | **Age matched reference values** |
| --- | --- | --- | --- | --- |
| **Age** | **10 years** |  | **2 years** |  |
| Hb (g/dL) | **10** | 11-13 | **9.8** | 10 -13 |
| MCV (fL) | **54** | 74-88 | **54** | 71 - 85 |
| Reticulocyte count (x10E9/L) | **36** | 42-70 | 40 | 36 - 68 |
| Transferrin saturation (%) | **73** | 20-45 | **70** | 20-45 |
| Iron (µg/dL) | 27 | 10-30 | 30 | 10-30 |
| Ferritin (µg/L) | **821** | 30-240 | **989** | 30-240 |
| Transferrin (mg/dL) | **150** | 203-360 | **170** | 200-350 |
| Hepcidin (nmol/L) | 1.5 | 0.5-14.7 | Not done |  |
| Total protoporphyrin µmol/L in erytroctes | 4.8 | <1.5 | 2.6 | <1.5 |
| Free protoporphyrin µmol/L, in erytroctes | 2.6 | <0.3 | 0.5 | <0.3 |
| Zink protoprophorin µmol/L, in erytroctes | 2.1 | <1.2 | 2.1 | <1.2 |
| Iron accumulation on MRI T2 liver (msec) | 3.3 | >8 | 4.7 | >8 |
| Iron accumulation on MRI T2 heart (msec) | 35 | >20 | Not done |  |

Altered values are outlined in bold.

**Supplementary Table S4.** Patients’ (P2.1 and P2.2) immunoglobulin levels in serum.

| **Patient** | **P2.1** | | **P2.2** |
| --- | --- | --- | --- |
| **Age** | **8 years** | **10 years*** | **4 years**** |
| Serum IgG | 540 (443-1.095) | **660** (700-1.500) | **480 (**500 - 1.300) |
| Serum IgA | 107 (36 -204) | 106 (45-395) | 31 (25 - 154) |
| Serum IgM | 72 (34 -190) | 75 (40-180) | 127 (30 - 180) |

Ig: immunoglobulin, units are done in mg/dL. Values in parenthesis correspond to age matched reference values. Altered values are outlined in bold. *IgG levels increased to 730 mg/dL at 10 years 9 months;**IgG levels increased to 550 mg/dL at 4 years 9 months, and although levels increased with age^7^, they consistently remained slightly below the reference values for their age group

**Supplementary Table S5. Lymphocyte immunophenotypic profile** **of P2.1 and P2.2**

| **Patient** | **P2.1 P2.2** | | |
| --- | --- | --- | --- |
| **Age** | | **5 years** | **4 years** |
| T-Cells | 3850 (700-4.200) | | 3170 (900-4.500) |
| CD4+T-Cells | | **2.280** (300-2.000) | 2.210 (500-2.400) |
| CD8+T-Cells | | 1.400 (300-1.800) | 720 (300-1.600) |
| B-Cells | | 730 (200-1.600) | 880 (200-2.100) |
| NK-Cells | | 390 (90-900) | **90** (100-1.000) |
| CD4/CD8 ratio | | 1,6 | 3,1 |

Units are done in cells/µL.Values in parenthesis correspond to age matched reference values. Altered values are outlined in bold

**Supplementary Table S6.** crRNAs, ssODNs and primers used in this study.

| **Variant** | **crRNA / ssODN (5'-3')** | **Strand** | **On-target score** | **Off-target score** | **Distance from DSB** | **Forward Primer (5'-3')** | **Reverse Primer (5'-3')** | **Result** | |
| --- | --- | --- | --- | --- | --- | --- | --- | --- | --- |
| **c.58T>C** | CTGAACCGGGTATATGACAA | + | 71 | 80 | 4 nt | AGAATGTTGGTTTGAAAGGAGAAC | ATGCTGAGCTGACATAACAGACTT | Successful |  |
|  | TATACCTAAACGAGCTTTTCCTAAACATTCCCGTGCCTGTTCTTCATCATGCCTTTTTCCGCAACACAGTTTGGTGGAGAACCATTGTCACATACCCGGTTCAGCCTGGCTCGGCAAGTAGATGGC |  |  |  |  |  |  |  |  |
| **c.713G>A** | AAACTGGTCCATGCTAATTT | + | 50 | 47 | 6 nt | AGCATGGGATAAAAGCATGAAAGA | TGGTATGAGAGTTTAAGATTGCTGA | Successful |  |
|  | TGTCTCAAGATTTTAGAGCTGTTCCGATACCATAATGCTCATTAAATCTAATGTCTTTGTTCTAGGGTAAACTGGTCCATGCTAATTTTGATACTAAAAAAGATTTTGAGGATTTATACACTCCTG |  |  |  |  |  |  |  |  |
| **c.1361G>A** | AAAGTCTCCAGCACTCCAAC | - | 82 | 53 | 4 nt | CCCCTGCTATGTACTCAAAATCTTA | GTAGCATTAGGGATTCATTTTCAA | Successful |  |
|  | ACTTTTTTCTCTTTCAGATGGGTTTCAGCCCAGCAGAAGCATTATCTTTGCAAGTTGGAATGCTGGAGACTTTGGATCGGTTGGTGCCACTGAATGGCTAGAGGTATTCTTTATCATCCCTTCCC |  |  |  |  |  |  |  |  |
| ***c.941C>T (design 1)** | AAGGGAATCCAGGTGTGTAA | - | 63 | 38 | 15 nt | AGTACTTTGTCAATAGTGCCTTGA | AACCATCCTCCAAATTCCCAAATG | Unsuccessful | |
|  | AAGTTTTGAGGAAGTATGTGATGGAGTGAATTGCTCTTTTGTTTTCCTAGGCTCATCTGGGGACAGGTGATCCTTACACACCTGGATTCCTTTCCTTCAATCACACTCAGTTTCCACCATCTCGGT |  |  |  |  |  |  |  |  |
| ***c.941C>T (design 2)** | ACTGAGTGTGATTGAAGGAA | - | 62 | 0 | 4 nt | AGTACTTTGTCAATAGTGCCTTGA | AACCATCCTCCAAATTCCCAAATG | Unsuccessful | |
|  | TTTGAGGAAGTATGTGATGGAGTGAATTGCTCTTTTGTTTTCCTAGGCTCATCTGGGGACAGGTGACCCTTACACACCTGGATTTCTTTCCTTCAATCACACTCAGTTTCCACCATCTCGGT |  |  |  |  |  |  |  |  |
| ***c.941C>T (design 3)** | AACTGAGTGTGATTGAAGGA | - | 78 | 0 | 5 nt | AGTACTTTGTCAATAGTGCCTTGA | AACCATCCTCCAAATTCCCAAATG | Unsuccessful | |
|  | TGAGGAAGTATGTGATGGAGTGAATTGCTCTTTTGTTTTCCTAGGCTCATCTGGGGACAGGTGACCCTTACACACCTGGATTCCTTTCCTTCAATCACACTCAGTTTCCACCATCTCGGT |  |  |  |  |  |  |  |  |

***** Unfortunately, after several attempts using multiple sgRNAs designs to target the *TFRC* c.941C genomic position, we were unable to obtain the KI model for this variant. A plausible explanation is that the genomic context of c.941C>T variant results in poor sgRNA performance, which decreases homology-directed repair efficiency.

**Supplementary References.**

1. Olbrich T, Mayor-Ruiz C, Vega-Sendino M, Gomez C, Ortega S, Ruiz S, et al. A p53-dependent response limits the viability of mammalian haploid cells. Proc Natl Acad Sci U S A. 2017;114(35):9367–72.
2. Beigl TB, Kjosås I, Seljeseth E, Glomnes N, Aksnes H. Efficient and crucial quality control of HAP1 cell ploidy status. Biol Open. 2020;9(11).
3. Muñoz‐Pujol G, Ugarteburu O, Segur‐Bailach E, Moliner S, Jurado S, Garrabou G, et al. CRISPR/Cas9-based functional genomics strategy to decipher the pathogenicity of genetic variants in inherited metabolic disorders. J Inherit Metab Dis. 2023;46(6), 1029-1042.
4. Holmes-Hampton GP, Ghosh MC, Rouault TA. Methods for Studying Iron Regulatory Protein 1: An Important Protein in Human Iron Metabolism. Methods Enzymol. 2018;599:139–55.
5. Tong WH, Rouault TA. Functions of mitochondrial ISCU and cytosolic ISCU in mammalian iron-sulfur cluster biogenesis and iron homeostasis. Cell Metab. 2006;3(3):199–210.
6. Colucci S. Abstract Book for the 27th Congress of the European Hematology Association. Hemasphere [Internet]. 2022;6:354–355. Available from: <https://onlinelibrary.wiley.com/doi/10.1097/01.HS9.0000852292.38263.b8>

7. Blanco E, Perez-Andres M, Arriba-Mendez S, Contreras-Sanfeliciano T,

Criado I, Pelak O et al. Age-associated distribution of normal B-cell and plasma

cell subsets in peripheral blood. J Allergy Clin Immunol. 2018;141:2208-2219
